# Passive degassing of lithospheric volatiles recorded in shallow young groundwater

**DOI:** 10.1038/s41561-025-01702-7

**Published:** 2025-06-05

**Authors:** R. L. Tyne, M. W. Broadley, D. V. Bekaert, P. H. Barry, O. Warr, J. B. Langman, I. Musan, W. J. Jenkins, A. M. Seltzer

**Affiliations:** 1 Department of Marine Chemistry and Geochemistry, Woods Hole Oceanographic Institution, Woods Hole, MA USA; 2 Department of Earth and Environmental Science, University of Manchester, Manchester, UK; 3 Centre de Recherches Pétrographiques et Géochimiques, CNRS UMR 7358, Université de Lorraine, Vandoeuvre-lès-Nancy, France; 4 Department of Earth and Environmental Sciences, University of Ottawa, Ottawa, Ontario Canada; 5 Department of Earth and Spatial Sciences, University of Idaho, Moscow, ID USA

**Keywords:** Hydrogeology, Solid Earth sciences, Geochemistry

## Abstract

The development of life on Earth has been enabled by its volatile-rich surface. The volatile budget of Earth’s surface is controlled by the balance between ingassing (for example, via subduction) and outgassing (for example, through magmatic and tectonic processes). Although volatiles within Earth’s interior are relatively depleted compared to CI chondrites, the total amount of volatiles within Earth is still substantial due to its vast size. However, the relative extent of diffuse degassing from Earth’s interior, not directly related to volcanism, is not well constrained. Here we use dissolved helium and high-precision argon isotopes combined with radiocarbon of dissolved inorganic carbon in groundwater from the Columbia Plateau Regional Aquifer (Washington and Idaho, USA). We identify mantle and crustal volatile sources and quantify their fluxes to the surface. Excess helium and argon in the groundwater indicate a mixture of sub-continental lithospheric mantle and crustal sources, suggesting that passive degassing of the sub-continental lithospheric mantle may be an important, yet previously unrecognized, outgassing process. This finding that considerable outgassing may occur even in volcanically quiescent parts of the crust is essential for quantifying the long-term global volatile mass balance.

## Abstract (web summary)

The sub-continental lithospheric mantle passively degasses volatiles to Earth’s surface, including from regions unaffected by volcanism, according to a study of helium and argon isotopes in young groundwater.

## Main

While volcanic degassing has canonically been considered the primary source of outgassing from our planet’s interior, recent studies have challenged this^1^. In particular the sub-continental lithospheric mantle (SCLM), which, while only comprising ~2.5% of the total mantle^2^, may provide a considerable juncture for devolatilization due to its adjacency to crustal settings. However, to date, this prospective outgassing pathway remains uncertain. Typically, continental volatile fluxes have been determined by the measurement of He isotopes in groundwater and springs^3–7^. However, there is often ambiguity in attributing volatile signals to sources because ^4^He is produced (from U and Th decay) within aquifers, in the deeper crust and in the mantle^3,4,8^. Additionally, different mantle sources have distinct ^3^He/^4^He compositions, which complicates the identification and quantification of mantle He inputs (for example, refs. ^8–12^). Argon-40, a radiogenic nuclide and the third most abundant constituent of the atmosphere, is a promising complementary tracer to He isotopes for the outgassing of other volatiles due to its inert nature and continual production (via K decay) within the mantle and crust^13–15^.

The majority of groundwater within the upper kilometre of the crust has a sufficiently long residence time (that is, the average amount of time since a parcel of water has been isolated from the atmosphere) to represent a useful archive of hydrogeologic processes and volatile fluxes^16,17^. Determination of excess (that is, non-atmospheric) dissolved He and Ar in aquifers offers the potential to provide insights into the transfers of volatiles between deep Earth and the surface. However, analytical limitations have largely precluded detection of excess ^40^Ar (^40^Ar*) in order ten thousand-year-old (ka) groundwater, due to the large atmospheric contribution. Deep radiogenic ^4^He and ^40^Ar fluxes have previously been observed in several ancient (>100 ka) waters from deep mines and artesian systems, where ^40^Ar/^36^Ar has been observed to exceed atmospheric ratios at the percent scale^14,18,19^. Recent developments for the analysis of heavy noble gas isotopes at sub-per-mille precision^20,21^ provides the quantitative resolution for robustly determining ^40^Ar* in younger groundwater through the ‘triple Ar isotope’ approach. This method uses the non-radiogenic Ar isotopes (^38^Ar and ^36^Ar) to disentangle atmospheric ^40^Ar from low-level geological input from the radioactive decay of K in the solid Earth^22,23^.

**Fig. 1:**
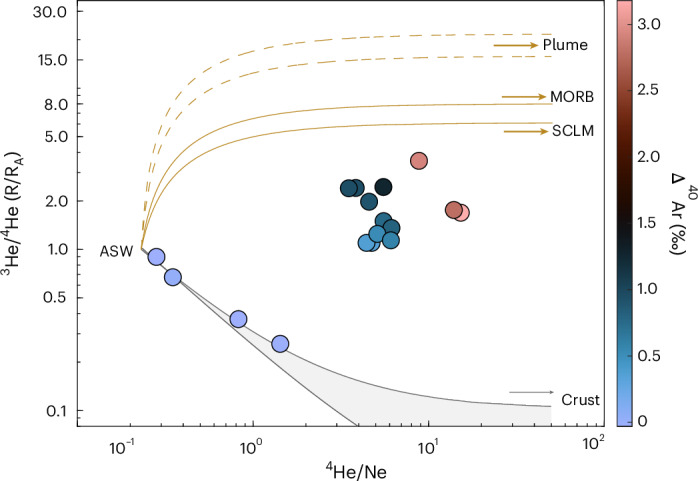
^3^He/^4^He vs He/Ne of the Palouse Basin Aquifer samples. Symbol colours correspond to the Δ^40^Ar excess (excess readiogenic ^40^Ar relative to atmospheric air) observed within the samples (*n* = 17), errors are *measured value* ±1σ uncertainties and are within the symbol size. The grey line represents mixing between ASW and crust (0.02 to 0.1 *R*_A_ (refs. ^30,31^)). The yellow lines represents mixing between ASW and mantle (SCLM 6.1 ± 2.1 R_A_ (ref. ^9^, where R_A_ is atmospheric ratio), MORB 8 ± 1 R_A_ (ref. ^8^) and deep mantle plume source 16–22 R_A_ (refs. ^10–12^)) (dashed lines). Four samples (Moscow 2, Moscow 3, Elk Golf and Parker Farm) are consistent with a purely crustal line; these samples also have no Δ^40^Ar excess and are referred to as the ‘crustal samples’. Source data

**Extended Data Fig. 1:**
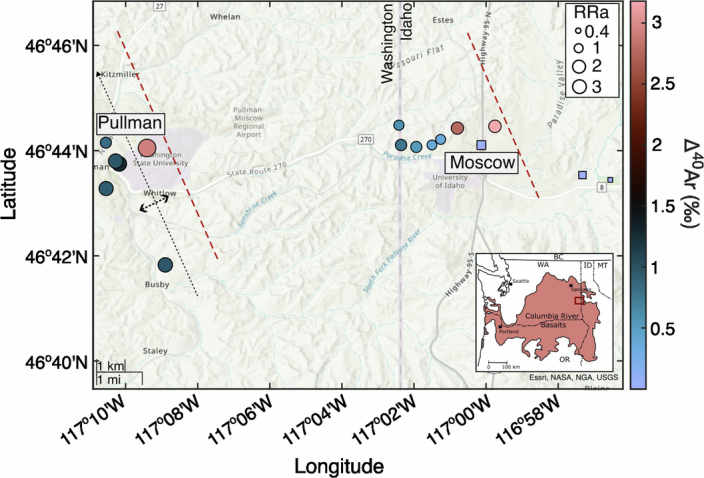
Map of study area showing the locations of the groundwater wells (n = 17) within the Palouse Basin Aquifer. The colour of the symbol relates to the excess ^40^Ar (Δ^40^Ar) and symbol size represents the ^3^He/^4^He of the sample relative to air (R/R_A_). Samples with the highest Δ^40^Ar correlate with the highest measured ^3^He/^4^He. The “crustal” samples (Main text) are shown with a square symbol. Red dashed lines are the Moscow and South Fork fault, and the black dashed line represents a plunging anticline. The inset in the bottom right shows the location of the study area (red rectangle) within the Columbia River Basalts with the main urban areas labelled. Basemap is from ESSRI ArcGIS (https://www.arcgis.com/apps/mapviewer/index.html). Source data

Here we report measurements of triple-argon isotopes and He isotopes (*n* = 33 and *n* = 21, respectively) in groundwater from 17 wells, alongside noble gas abundances and radiocarbon activity of dissolved inorganic carbon (DIC)^24^ (Extended Data Fig. 1), to determine deep volatile sources and fluxes to the Palouse Basin Aquifer (PBA). Physiochemical parameters and associated well depths can be found in Extended Data Table 1. The PBA is a fractured-rock and interbedded sediment aquifer system that supplies municipal water to regional communities as part of the Columbia Plateau Regional Aquifer (CPRA). This CPRA comprises several units of the Columbia River Basalt Group (CRBG) that probably formed as a result of the Yellowstone hotspot (Methods). Previous work within the study area has suggested the presence of mantle carbon input to deep groundwater^25^, however the origin, migration pathway and timing of this inferred mantle input is unknown. This investigation integrates He isotope, radiocarbon and high-precision triple-argon-isotope measurements and proposes a multi-tracer approach towards utilizing palaeogroundwater as a record of volatile input from deep crustal and mantle sources to Earth’s surface. The addition of the ^40^Ar tracer alongside helium isotopes allows for both a mantle contribution and the source of this contribution (for example, in situ vs ex situ) to be identified, which was not previously possible with the helium isotope alone, as the intermediate ^3^He/^4^He are between various mantle endmembers and the crust (Fig. 1), complicating any attempts to evaluate the mantle source.

## Noble gas excesses in groundwater

**Extended Data Fig. 2:**
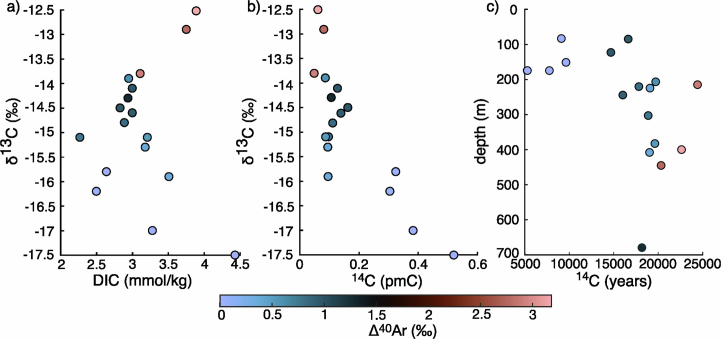
Carbon and Radiocarbon data from the groundwater samples. Symbol colour correlates to the Δ^40^Ar of each groundwater sample (n = 17). **a**) The δ^13^C of dissolved oxygen concentration (DIC) as a function of DIC concentration. There is no correlation between δ^13^C and concentration. **b**) The δ^13^C of DIC vs radiocarbon in percent modern carbon (pmC). **c**) depth of groundwater well vs radiocarbon residence time (years). The four crustal samples identified in Fig. 2 are the shallow samples with residence times <10,000years and lowest Δ^40^Ar. Source data

Radiocarbon activities (of DIC) are between 3.5 and 52.0 percent modern carbon (pmC), corresponding to apparent groundwater residence times between ~27,000 and ~9,000 years. These ages are in agreement with previous studies in the area^25,26^—although the oldest ^14^C ages may exhibit small biases (order 1 ka) due to ^14^C-free mantle carbon input (Extended Data Fig. 2 and Methods). Concentrations of Ne, Ar, Kr and Xe are in agreement with predictions from the expected noble gas concentrations in groundwater due to equilibrium gas exchange and excess air dissolution (closed equilibrium (CE) model, see ref. ^27^) at 800 m surface elevation (0.91 atm) with temperatures between 4.7 and 8.1 °C (ref. ^24^) and an excess air component. However, He concentrations vary by nearly two orders of magnitude from 0.08 to 4.44 × 10^−6^ cm^3^_STP_ g_w_^−1^, where STP is standard temperature and pressure and w is water (Extended Data Tables 2 and 3). Similarly, measured ^3^He/^4^He (R), reported as R/R_A_ relative to the atmospheric ratio (R_A_), range between 0.24 and 3.37 R_A_. Surprisingly, both the highest ^4^He concentrations and the highest ^3^He/^4^He are observed in the deepest (oldest) samples, suggesting that deep volatile sources contribute mantle and radiogenic He to the aquifer. This result is remarkable, because, outside of a direct volcanic setting, the conventional expectation is for groundwater to inherit crustal ^4^He from radioactive decay of U and Th in aquifer minerals, leading to a lowering of ^3^He/^4^He with increasing ^4^He.

Helium in groundwater is derived from a combination of atmospheric, mantle and crustal sources. Combining ^3^He/^4^He with ^4^He/Ne is a useful approach to identify and distinguish distinct He sources^28,29^, because, unlike ^4^He, Ne only has an appreciable atmospheric source. In the PBA, there is a considerable mantle He contribution in groundwater from 13 of the 17 wells (Fig. 1). The remaining four wells (Elk Golf, Parker Farm, Moscow 2 and Moscow 3) have excess helium (that is, ^4^He/Ne above air-saturated water (ASW)) but have no discernible mantle contribution, as they lie along a mixing curve between ASW and a pure crustal endmember (^3^He/^4^He ~ 0.1 R_A_ (refs. ^30,31^)). These four dominantly crustal samples (hereafter, ‘crustal samples’) have the youngest radiocarbon ages (<10,000 years) and the warmest recharge temperatures (>7 °C) (refs. ^24^). These crustal samples were collected from shallow wells (<175 m) in the east of the study area (Extended Data Fig. 1), where recent, local recharge is known to occur^26,32^ resulting in no discernible deep mantle flux in these samples. Samples with the highest ^3^He/^4^He—indicative of the greatest mantle contributions—are from the deep wells in the western part of the study area (Methods).

To quantify He excesses (^3^He_xs_ and ^4^He_xs_) in groundwater, atmosphere-derived He (that is, the component from air–water equilibration and excess air determined using the CE model) is subtracted from measured ^3^He and ^4^He concentrations (Methods). Modelled atmospheric (CE model) He isotope concentrations can be found in ref. ^24^. Excess ^4^He concentrations vary between 0.02 and 4.38 × 10^−6^ cm^3^_STP_ g_w_^−1^ and correlate with ^3^He_xs_, which is up to 1.09 ± 0.01 × 10^−11^ cm^3^_STP_ g_w_^−1^.

**Extended Data Fig. 3:**
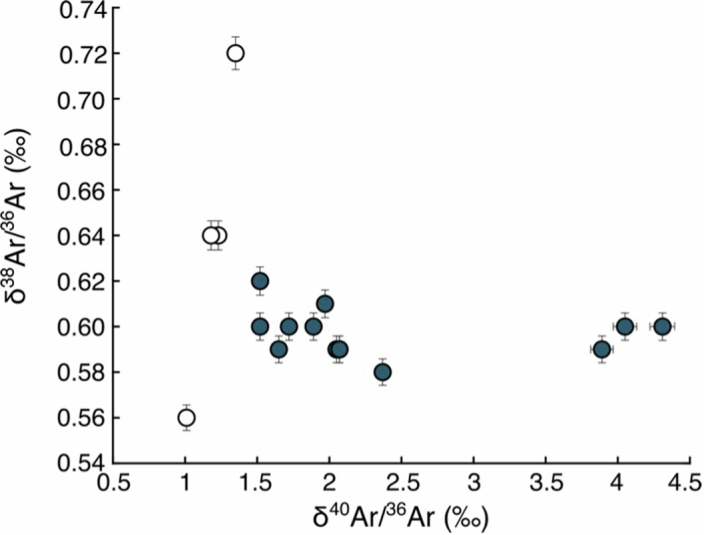
Triple Argon Plot (δ^40^Ar/^36^Ar vs δ^38^Ar/^36^Ar) for the groundwater samples (n = 17). *Mean* ± 1σ uncertainties shown, where no errors are shown these are within symbol size. Unfilled samples represent the four “Crustal” samples (Main text). There is no correlation between the two parameters and therefore changes in δ^40^Ar/^36^Ar are not a result of fractionation. Source data

**Extended Data Fig. 4:**
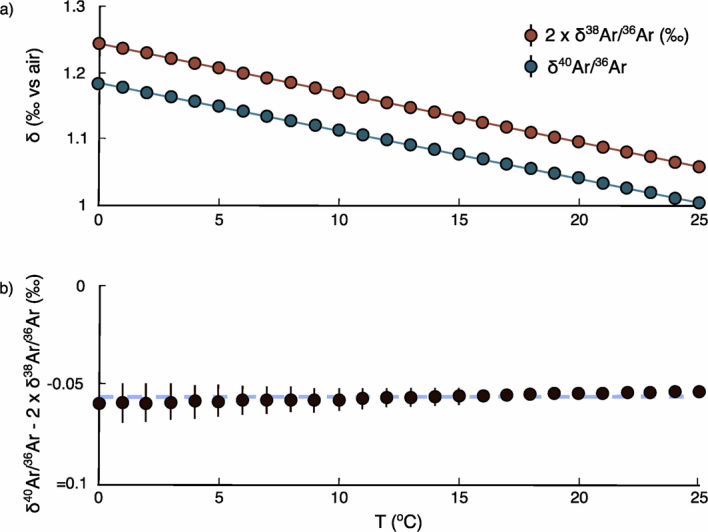
Deviations and mass fractionation of Argon isotopes in Air Saturated Water relative to air. Air saturated waters (n = 26) were measured at 1^o^C intervals from 1 to 25^o^C. *Measured value* ±1σ uncertainties are shown. **a**) δ^40^Ar/^36^Ar and 2 x δ^38^Ar/^36^Ar in air-saturated water between 0 and 25^o^C. There is an approximately constant offset in fresh water between 0 and 25 °C according to the updated solubility functions based on recent air-water equilibration experiments^61^. **b**) The offset between δ^40^Ar/^36^Ar and 2 x δ^38^Ar/^36^Ar at each temperature. The mean offset over this temperature range is shown by the blue dashed line and is calculated to be 0.057‰ which is adopted in the updated definition of Δ^40^Ar. Source data

The excess radiogenic Ar can also be investigated relative to atmospheric air (Δ^40^Ar) in per mille (Methods and Extended Data Figs. 3 and 4). While the highest Δ^40^Ar values were identified in the oldest samples (that is, samples with the lowest radiocarbon activities), the four dominantly crustal samples (Fig. 1) do not exhibit any discernible ^40^Ar*. Notably, the highest measured radiogenic ^40^Ar excess (Δ^40^Ar = 3.18 ± 0.02‰; ^40^Ar* = 1.36 × 10^−6^ ± 0.01 cm^3^_STP_ g_w_^−1^) would not be discernible from air or ASW at the precision of static noble gas mass spectrometry (that is, ~5‰ vs ~0.01‰ for dynamic mass spectrometry), highlighting the importance of this new technique to expand the application of radiogenic Ar isotope analyses to a wider range of natural samples.

**Fig. 2:**
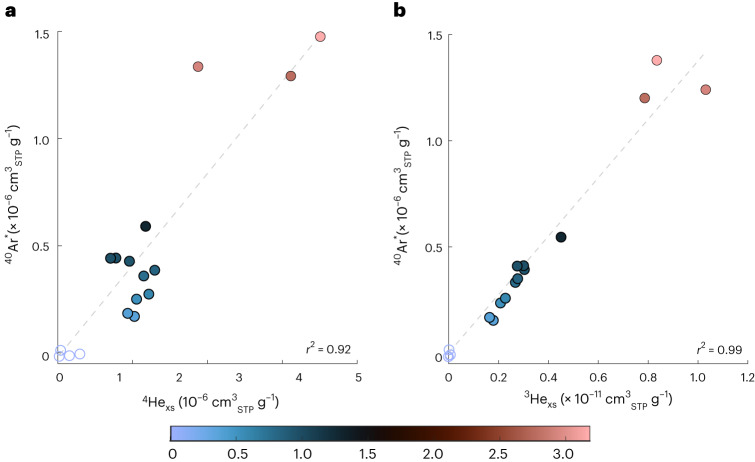
Relationship between ^4^He_xs_ and ^3^He_xs_ and concentrations relative to ^40^Ar* in the Palouse Basin Aquifer samples. **a**,**b**, Relationship between ^4^He_xs_ (**a**) and ^3^He_xs_ (**b**) and concentrations relative to ^40^Ar* in the Palouse Basin Aquifer samples (*n* = 17). Symbol sizes are larger than the *measured excess* ±1*σ* uncertainties. Unfilled = samples with only crustal noble gas addition from in situ production. Dashed lines represent the line of best fit through the samples with the external excesses (filled) and the *r*^2^ value for each is shown in the bottom right corner (note: unfilled ‘in situ only’ samples were not used in calculating the correlation). Source data

We observe a striking correlation between ^4^He_xs_ (measured via static mass spectrometry) and ^40^Ar* (measured via dynamic mass spectrometry) (Fig. 2; *r*^2^ = 0.92), indicating that their accumulations are related. Surprisingly, an even stronger correlation (*r*^2^ = 0.99) exists between ^3^He_ex_ and ^40^Ar*. Excess ^3^He is predominately derived from the mantle, and its production within the PBA^33^ and from the decay of tritium should be negligible (Methods), therefore this high correlation may indicate that a substantial portion of ^40^Ar* may also derive form the mantle. We note that per-mille-scale Δ^40^Ar anomalies were also previously measured in ~10,000-year-old groundwater in Southern California, in the first application of this new technique^23^. At the time, it was speculated that weathering of aquifer minerals represented the likely ^40^Ar release mechanism into groundwater. However, the unexpectedly strong correlation between ^3^He and ^40^Ar observed here (and the lack of He isotope measurements in the previous study) raises the possibility that elevated ^40^Ar* identified in Southern California groundwater may likewise reflect an input of mantle-derived volatiles, perhaps in relation to the nearby San Andreas fault^7^. Similarly, in the Tucson Basin, where ^3^He measurements indicate no mantle contribution, ^40^Ar* was consistently found to be zero (within error) in groundwaters (up to 30,000 years) (ref. ^20^). The lack of excess ^40^Ar in a system without mantle input further hints at the mantle playing a potentially dominant role in the flux of ^40^Ar to shallow groundwater. Diffusive degassing of mantle volatiles has important implications for the use of radiogenic volatiles as groundwater residence time tracers, as ^4^He is a common groundwater dating tool. Thus, the notion that ^40^Ar (like ^3^He and ^4^He) has a mantle source in some shallow groundwater settings raises the possibility that ^40^Ar may offer additional constraints to refine and improve ^4^He dating.

In this study, our focus is on the origin of deep volatiles, and we dedicate the following analysis to better understand (1) how mantle-derived ^40^Ar infiltrates aquifers and (2) how important this mantle flux may be within the framework of the global volatile cycle.

## Origin of gases in the Columbia Plateau regional aquifer

The observation of substantial ^4^He_xs_, ^3^He_xs_ and ^40^Ar* in PBA groundwater, which are all correlated, suggests a co-genetic relationship between the geological sources of noble gases within the system. ^4^He_xs_ and ^40^Ar* could accumulate either because of (1) in situ production from the decay of U, Th and K decay in the aquifer minerals and subsequent release into the groundwater via diffusion and/or mineral dissolution or (2) an external, deeper flux from a crustal and/or mantle source. The presence of mantle-derived ^3^He within the aquifer suggests a mantle-derived volatile flux into the system.

Here we determine the amount of external He input (^3^He_ext_ and ^4^He_ext_) from deep crustal and mantle sources, below the PBA, by subtracting in situ He isotope production from ^3^He_xs_ and ^4^He_xs_ (Methods). Assuming that the crustal dominated samples have no external contributions, these concentrations can be used to generate conservative estimates of ^3^He_ext_ and ^4^He_ext_ to be between 1.6 and 10.8 × 10^−12^ cm^3^_STP_ g_w_^−1^ and between 0.61 and 4.05 × 10^−6^ cm^3^_STP _g_w_^−1^, respectively. Assuming that all ^4^He_xs_ in these samples is derived from in situ production, the in situ accumulation rate of ^4^He (and ^3^He) in each sample can be calculated from ^4^He_xs_ concentrations, radiocarbon ages and a crustal production endmember of 0.1 Ra. The average in situ accumulation rate for the crustal samples is 1.49 × 10^−11 ^cm^3^_STP_ g_w_^−1^ yr^−1^, similar to previous estimates of in situ ^4^He production within the CRBGs (1.7 × 10^−11^ cm^3^_STP_ g_w_^−1^ yr^−1^ (ref. ^34^)). In the 13 mantle-influenced wells, we then apply these estimated in situ accumulation rates and, using the radiocarbon ages, we quantify the amounts of excess ^4^He and ^3^He that has resulted from in situ production, using a Monte Carlo approach to propagate uncertainties (Methods).

**Fig. 3:**
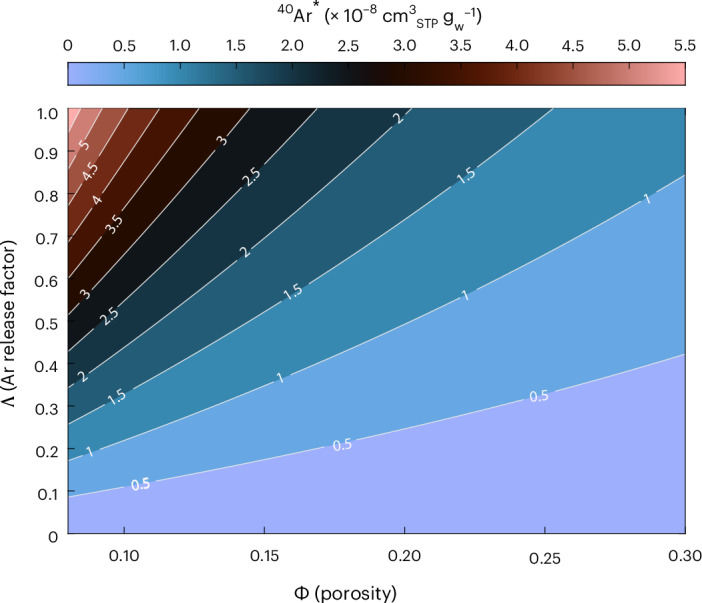
Modelled concentration of ^40^Ar* from in situ production and release over 20,000 years within the CPRA. ^40^Ar* concentrations are modelled for varying release factors (0–1) and porosities (0.08–0.3 (ref. ^35^)). White lines and numbers represent the contours of ^40^Ar* concentration (× 10^−8 ^cm^3^_STP_ g_w_^−1^) output from the model. Expected ^40^Ar* concentration is calculated following the methods in ref. ^35^. The maximum concentration of ^40^Ar* produced in situ is ~5.5 × 10^−8^ cm^3^_STP_ g_w_^−1^. Source data

In principle, we can similarly quantify the in situ production and accumulation of ^40^Ar to estimate external ^40^Ar (^40^Ar_ext_). While it is conventionally assumed that all He produced within aquifer minerals readily diffuses into groundwater (that is, release factor ~ 1; ref. ^30^), the limited work on ^40^Ar* in groundwater suggests that low-temperature diffusive release of ^40^Ar* from a mineral is much slower than He, owing to the larger atomic radius of Ar^14,23,33^. To estimate the maximum amount of ^40^Ar* from in situ production within the PBA, we follow the methods of ref. ^35^ (Methods), assuming a maximum K concentration of 2.3 wt% (ref. ^36^). We then model in situ ^40^Ar* production across a wide range of release factors (from 0 to 1 (ref. ^37^), given the lack of deformation and stability in the PBA) and across the published range of porosity values in the CPRA (from 0.08 to 0.3 (ref. ^36^)). We find that even with a release of 1 (that is, 100% release) and a minimum porosity of 0.08, the maximum amount of ^40^Ar* that can accumulate over ~20,000 years (~5.5 × 10^8^ cm^3^_STP_ g_w_^−1^) represents, at most, only 4% of the highest ^40^Ar* observed in this study (1.36 × 10^−6^ cm^3^_STP_^−1^ g_w_^−1^) (Fig. 3). Adopting the most plausible parameters (for example, a release factor <<1 (ref. ^14^) and a median porosity of ~0.2), the vast majority of ^40^Ar^*^ in all samples must be derived from an external source, with only negligible input of ^40^Ar to the aquifer from in situ production within the CPRA minerals (that is, ^40^Ar_ext_ ≈ ^40^Ar*).

**Fig. 4:**
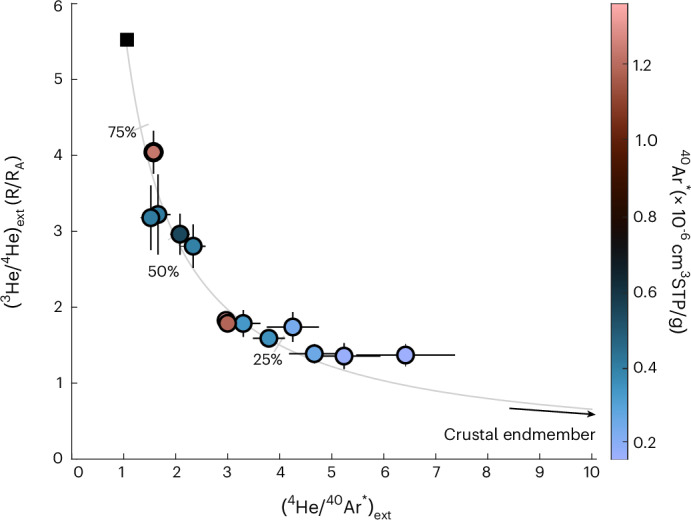
Relationship between external ^3^He, ^4^He and ^40^Ar excesses within the groundwater samples that have a mantle contribution. ^3^He/^4^He_ext_ vs ^4^He/^40^Ar*_ext_ from the mantle-influenced samples (*n* = 13) is used to model the expected mixing between mantle and crustal endmembers (grey line). The black square represents the most likely mantle composition (SCLM) based on a ^4^He/^40^Ar* within the mantle range (2 ± 1 (ref. ^8^)). Tick marks represent a 75%, 50% and 25% mantle contribution. The crustal ^4^He/^40^Ar* is probably highly fractionated, but changing this value only has minimal effect on the mantle endmember composition. Symbol colours represent the ^40^Ar* concentration within the samples and *external values* ± 1*σ* uncertainties are shown. The four dominantly crustal samples high the highest ^4^He/^40^Ar*_ext_ and lowest ^3^He/^4^He_ext_. Source data

**Extended Data Fig. 5:**
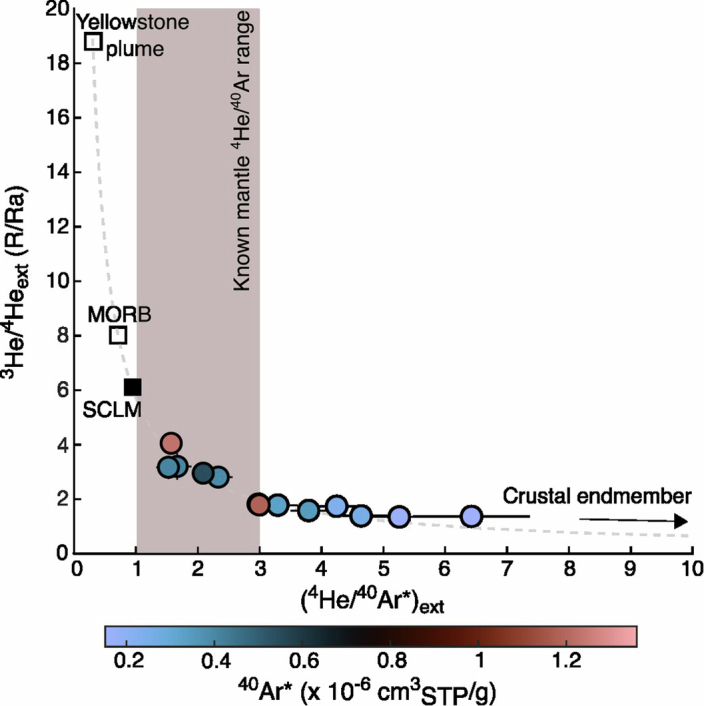
Mixing between potential mantle endmembers and crust for external ^3^He, ^4^He and ^40^Ar excess. ^3^He/^4^He_ext_ vs ^4^He/^40^Ar*_ext_ from the mantle influenced samples (n = 13) is used to model the expected mixing between a modelled mantle endmember and a crustal endmember (Grey dashed line). Measured values are given with 1σ uncertainties. The crustal ^4^He/^40^Ar* is likely highly fractionated and changing the value has minimal effect on the mantle endmember composition. The black square represents the most likely mantle composition (SCLM) based on a ^4^He/^40^Ar* within the mantle range (2 ± 1^8^). The unfilled black squares represent the known Mid Ocean Ridge Basalt (MORB) and Yellowstone plume helium isotope ratio values^8,10–12^). Associated ^4^He/^40^Ar are significantly lower than the known mantle range for both MORB and the Yellowstone Plume and are unlikely to be the source of the mantle-derived fluids within the system. Source data

Understanding the source of this external flux is important for quantifying global volatile fluxes from diffuse degassing, and aquifers represent an excellent tool to constrain the flux. The highest observed (^3^He/^4^He)_ext_ of 4.0 ± 1.4 R_A_ coincides with a (^4^He/^40^Ar)_ext_ of ~1.6 (Fig. 4), both of which fall within the ranges of values characteristic of the SCLM (that is, 6.1 ± 2.1 R_A_ (ref. ^9^) and 1–3 (ref. ^8^), respectively), suggesting that the SCLM is the main volatile source. It is however possible that these values could be a co-incidental mix of a higher mid-ocean-ridge basalt (MORB)/plume endmember and the deep continental crust. To further distinguish between mantle volatile sources, we constructed a mixing model using the calculated externally derived concentrations of He and Ar isotopes (^3^He_ext_, ^4^He_ext_ and ^40^Ar*) and assuming all ^40^Ar* is externally sourced (as discussed above) (Fig. 4 and Extended Data Fig. 5). Our data closely plot along a mixing curve between a high ^3^He/^4^He_ext_ and low ^4^He/^40^Ar* endmember (mantle derived) and a low ^3^He/^4^He_ext_ and high ^4^He/^40^Ar* endmember (crustal derived). We adopt a Monte Carlo least squares fitting approach to determine the composition of the crustal and mantle endmembers (Methods). We find that a high crustal ^4^He/^40^Ar endmember (>100) is required, suggesting that the crust-derived ^4^He/^40^Ar in the system has been highly fractionated (that is, enriched in ^4^He) compared to typical crustal ^4^He/^40^Ar production ratios (~4 (refs. ^15,18,30^)). We suggest that the inferred high ^4^He/^40^Ar endmember reflects the preferential release of ^4^He relative to ^40^Ar* from a deeper crustal source, due to the higher release temperature of Ar from minerals^30^. Notably, similar fractionation in the ^4^He/^40^Ar* (>100) has been observed in other groundwater studies worldwide^13,38^. The same fractionation is not expected within the mantle endmember, due to the higher temperatures allowing for quantitative release of both ^4^He and ^40^Ar*, unlike the crustal source.

Our Monte Carlo mixing model approach enables estimation of the mantle source ^3^He/^4^He by adopting an assumed mantle ^4^He/^40^Ar of 1 (that is, the lowest end of the canonical range^8^) and evaluating the mixing curve at this value after fully propagating uncertainties in both ^3^He/^4^He_ext_ and ^4^He/^40^Ar_ext_ (Methods). This yields an implied mantle source ^3^He/^4^He of 5.5 ± 0.4 R_A_, consistent with the He isotope composition of the SCLM^9^, but statistically incongruent with either a MORB or plume-like mantle helium source. We note the mantle ^4^He/^40^Ar* of the mantle endmember may be fractionated by degassing or during transport (Methods). Although there are two possible minor tectonic faults with minimal offset within the study region (Extended Data Fig. 1), the basin is considered stable and lacks deformation^39–41^, and as a result, we do not expect much influence from movement along faults within the PBA. We suggest that the conceptually most straightforward and likely source of mantle-derived volatiles to the PBA is diffusive degassing of the SCLM. Furthermore, the setting of the PBA, far inland from the Cascadia subduction zone and distal (>500 km) from the current location of the Yellowstone hotspot with no thermal evidence of mantle upwelling^42^ (Methods), further supports the suggestion of a SCLM source.

## Implications for global volatile fluxes

The findings of this multi-tracer study suggest that passive ^40^Ar (and He) degassing from the SCLM through shallow groundwaters may represent a broader, yet underappreciated, mechanism for large-scale degassing of related mantle volatiles including CO_2_, nitrogen and sulfur^43,44^ to the upper crust and atmosphere. This work demonstrates a previously hidden volatile flux from the mantle that can accumulate appreciably, even in relatively young groundwater systems. The results of this study imply that the contribution of passive mantle outgassing to the global volatile balance may be more prevalent than previously considered. For example, understanding mantle and crustal fluxes has important implications for models of mantle and crustal outgassing and for long-term and large-scale geochemical evolution of the major terrestrial reservoirs (for example, atmosphere and mantle)^22,45,46^.

Future measurements made possible by the triple-argon-isotope approach will enable investigation of mantle ^40^Ar fluxes to groundwater on a broader scale. The recognition that diffuse volatile degassing from the SCLM through continental aquifers may be more ubiquitous than originally hypothesized^22,47,48^. Our findings of mantle He and Ar input into the shallow crustal settings reveals that evidence for hidden fluxes of mantle volatiles to the upper crust and atmosphere need to be considered when determining whether our planet is currently in net ingassing (that is, influx via subduction > outflux via degassing) or outgassing regime, which may have important impacts in terms of evaluating the role of long-term volatile cycling on terrestrial biogeochemical cycles.

## Methods

### Geological history

The Columbia River Basalt Group (CRBG) are flood basalts formed between 16.7 and 5.5 million years ago^49^. The flows originated from north–northwest-trending fissures in eastern Oregon, eastern Washington and western Idaho^50,51^. It has been proposed the eruption could have originated from the subduction related process such as slab tear^33^ or slab roll back^52^ or from the initiation of the Yellowstone hotspot plume^53–55^. The flows can be subdivided into seven formations (Steens, Imnaha, Grande Ronde, Picture Gorge, Prineville, Wanapum and Saddle Mountains basalts), which have different aerial extents over the basin and consistent of multiple lava flows^51^. Most flows have a columnar base as a result of slow cooling of ponded lava, which is overlain by irregular jointed basalt (entablature) and a vesicular and scoracious top that experienced more rapid cooling. The bedrock that underlies the Columbia River Basalt Group consists of pre-Miocene igneous, metamorphic and consolidated sedimentary rocks.

The Palouse Basin Aquifer is located on the eastern margin of the Columbia River Flood Basalt province and is contained in the mixed sediments of the Latah Formation and lava flows of the Columbia River Basalt Group^39^. This region is composed of 25 basalt flows that intruded into the basin from the west and disrupted westward drainages carrying eroded material from the basin mountains that are primarily composed of the granites of the Idaho Batholith. The sediments were captured between successive low-permeability basalt flows and alongside the higher-porosity zones of the basalts (such as the flow tops), representing recharge pathways from the eastern mountain fronts beyond the extent of the basalt. The interbedded sediments are clay rich, poorly sorted and are interspersed with coarse-grained channel deposits.

### Analytical techniques

#### Sample collection

To understand the different processes affecting groundwater residence time tracers, we collected samples from 17 groundwater samples from drinking water wells within the Palouse Basin Aquifer (Extended Data Fig. 1). Noble gases were collected in 3/8” Cu tubes and sealed using stainless steel clamps following standard procedures (for example, ref. ^56^). Approximately 3.5 l of water was collected for high-precision noble gas measurement following the procedures of ref. ^20^. Radiocarbon was collected in 100 ml glass bottles using standard procedures for groundwater outlined in ref. ^57^. Temperature pH and salinity were determined onsite using a Hanna HI98194 multiparameter meter. This work is part of a broader hydrogeological study, and here we report helium and argon isotopes for the first time, alongside measurements of radiocarbon of DIC and neon, argon, krypton and xenon abundances that have been published to an online repository^24^.

#### Radiocarbon

Radiocarbon and carbon isotopes of DIC were analysed at the National Ocean Science Accelerator Mass Spectrometry (NOSAMS) Laboratory at Woods Hole Oceanographic Institution (WHOI) following their standard protocols. Carbon-14 results are reported in percent modern carbon (pmC) and the reported analytical varied from 0.0012 to 0.0021 pmC.

We observe no relationship between in the *δ*^13^C_DIC_ (−12.9 to −17.0‰) and DIC concentrations (2.33 to 4.43 mmol kg^−1^) (Extended Data Fig. 2), suggesting there is no dead carbon input from the soils to the system, which is expected in basalt aquifers as they contain low amounts of inorganic and organic carbon^58,59^. As a result, we do not apply a correction for dead soil carbon input to our radiocarbon age. We do, however, expect that there is some fraction of mantle-derived carbon in the system that is radiocarbon dead based on prior work^25^. However, we note that the maximum observed δ^13^C is −12.5‰, which is far below the mantle value and closer to that of DIC in equilibrium with soil CO_2_ ( ~ −15‰). Even in an extreme scenario in which 50% of the carbon is from the mantle rather than the atmosphere, the impact on radiocarbon ages is limited to the half life of ^14^C, ~5,700 years, below the prescribed uncertainty in our Monte Carlo simulations.

#### Noble gases

Noble gas concentration and helium isotope ratios were measured within the Jenkins Laboratory at WHOI using a quadrupole mass spectrometer for noble gas abundances and a custom static mass spectrometer for helium isotopes^60^. Noble gases were initially extracted from Cu tubes, transferred into glass bulbs and cryogenically transferred and gettered through a fully automated system. Full procedures can be found at https://www2.whoi.edu/site/igffacility/analytical-capabilities-for-water-measurements/.

#### High-precision noble gases

Triple-argon isotopes were measured via dynamic dual inlet isotope ratio mass spectrometry in the Seltzer Laboratory at WHOI. A total of 33 samples (17 wells) were analysed, with a pooled standard deviation of 0.02‰ for δ^40^Ar/^36^Ar, 0.01‰ for δ^38^Ar/^36^Ar and 0.03 ‰ for Δ^40^Ar. Gases were equilibrated into the headspace of the space vessel on an orbital shaker (minimum three days) in an isothermal chamber before the water was drained, leaving behind ~100 ml (ref. ^20^). The headspace gases were then transferred and purified by gettering with titanium sponge at 900 °C to quantitatively remove all non-noble gases before cryogenically transferring the remaining noble gases (Ar, Kr and Xe) into a dual-valve dip tube^21^. After a minimum of three hours equilibration in a water bath at 30 °C, the sample was then attached to a custom Thermo MAT 253 Plus and analysed following the procedures in ref. ^21^. Following measurement, data were corrected for matrix effects, nonlinearity and low-mass tail interferences on ^36^Ar and ^38^Ar from ^40^Ar (ref. ^21^).

Δ^40^Ar is defined as excess radiogenic ^40^Ar, relative to atmospheric air, in per mille. The original definition of Δ^40^Ar was presented in ref. ^23^. After several dozen additional measurements of Ar isotope ratios in air–water equilibration experiments^61^, here we update the definition to reflect these new data. By definition, Δ^40^Ar must be equal to 0 for air-saturated water, and therefore, assuming mass-proportional fractionation, δ^40^Ar/^36^Ar − (2 × δ^38^Ar/^36^Ar) should equal 0 in ASW. However, 2 × δ^38^Ar/^36^Ar is consistently higher than δ^40^Ar/^36^Ar in air-saturated water based on our measurements (and validated by MD simulations) by an average of 0.057 per mille in the range of 0 to 25 °C (Extended Data Fig. 4; ref. ^61^). We therefore re-define Δ^40^Ar as:

1 $$\begin{array}{l}\Delta{40\atop}\mathrm{Ar}\,\left({{\permil}}\right)\,=\,{{40\atop}40{\mathrm{Ar}}*}\left/{40\atop}\mathrm{Ar}_{\mathrm{atm}}\,\times\,1,000\,\right.\\=\delta {40\atop}\mathrm{Ar}\left/{36\atop}\mathrm{Ar}\,-\,\left(2\,\times\delta {38\atop}\mathrm{Ar}\left/{36\atop}\mathrm{Ar}\right)\,+\,0.057\right.\right.\end{array}$$

where ^40^Ar* and ^40^Ar_atm_ refer to radiogenic (excess) and atmospheric concentrations of ^40^Ar, respectively, and δ refers to deviations in the Ar isotope ratios from the well-mixed atmosphere (in ‰).

Using this definition, and assuming all gas-phase Ar isotope fractionation (gravity, thermal diffusion, water vapour flux) is mass proportional, then between 0 °C and 25 °C, this formula robustly ensures that Δ^40^Ar will be zero for all samples with purely atmosphere-derived ^40^Ar that may be fractionated by physical processes. The concentration of excess ^40^Ar (that is, ^40^Ar*) may be calculated using ^40^Ar and a measurement of total Ar by noting that the total measured concentration of ^40^Ar (^40^Ar_tot_, cm^3^_STP_ g_w_^−1^) is equal to the sum of atmosphere-derived ^40^Ar (^40^Ar_atm_) and radiogenic ^40^Ar (^40^Ar):

2 $${\scriptstyle{40}\atop}\mathrm{Ar}* \,=\,{\scriptstyle{40}\atop}{\mathrm{Ar}}_{{\rm{tot}}}-\,{\scriptstyle{40}\atop}{\mathrm{Ar}}_{{\rm{atm}}}={\scriptstyle{40}\atop}{\mathrm{Ar}}_{{\rm{tot}}}\,\left/\,\left(1+1\left/\left(\Delta {\scriptstyle{40}\atop}\mathrm{Ar}\left/1,000\right)\right)\right.\right.\right.$$

Note that ^40^Ar_atm_ reflects contributions and physical fractionation of argon from equilibrium dissolution at the water table, fractionation in overlying soil air (for example, by gravity) and from excess air input, hence the need to utilize the triple Ar isotope composition (Δ^40^Ar) to fully account for the fractionated atmospheric component of ^40^Ar.

### Tritium in the Palouse Basin Aquifer

The decay of tritium ^3^H to ^3^He may result in an elevated ^3^He/^4^He in groundwater (for example, ref. ^17^). Although tritium was not measured as part of this study, previous measurements within region found very low levels of tritium indicate limited influence of very young groundwater and ruling it as a cause of elevated ^3^He (ref. ^62^).

Independent of this previous study, the scale of measured ^3^He_xs_ demonstrably exceeds any plausible amount of ^3^He production from tritium decay as reasoned below. First, we consider in situ production. In basaltic-based freshwater systems, a recent study estimated that 6.41 × 10^−4^ atoms of ^3^H per cm^3^ of fluid are generated annually^63^. When this is compared to the ^3^He content of ASW (Lake Baikal 10 °C), the ^3^He content represents 1.65 × 10^6^ atoms ^3^He per cm^3^ of fluid. Consequently, assuming all ^3^H decays to ^3^He, to increase the starting ^3^He content of ASW by just 1%, would take 25.8 million years—almost twice that of the maximum Columbia River Basalt Group age (16.7 million years)^49^. As an alternative scenario, we also consider an unrealistically extreme example where a considerable amount of 1950s groundwater is present (say, 100 TU (tritium units) at the time of groundwater recharge), if this had all decayed to ^3^He, we would expect to see ~2.5 × 10^−13^ cc_STP_ g^−1^ (100 TU / 4.021 × 10^14^ to convert to cc g^−1^) of tritigenic ^3^He. Such an amount of tritigenic ^3^He is 1–2 orders of magnitude below the excess ^3^He measured in our highest ^3^He/^4^He samples (which display excess ^3^He on the orders of 10^−11^ and × 10^−1^ cc_STP_ g^−1^). Given the low radiocarbon activities in these samples, this scenario can be ruled out, and the likely amount of tritigenic ^3^He is probably at least an order of magnitude smaller. As a result, we are confident that tritigenic ^3^He represents <1% of ^3^He excess in these samples.

### Isotopic source deconvolution and mixing analysis

In this section, we provide extra details on the source deconvolution of He and Ar isotopes.

Using measured data from each well (abundances of ^4^He, ^3^He, Ne, Ar, Kr and Xe, along with Δ^40^Ar), this deconvolution ultimately quantifies the sources of ^3^He, ^4^He and ^40^Ar.

The concentration of a noble gas isotope measured (${C}_\mathrm{i}^{\mathrm{meas}}$) represents the concentration inherited during atmospheric exchange during recharge (${C}_\mathrm{i}^{\mathrm{atm}}$) plus an excess contribution (${C}_\mathrm{i}^{\mathrm{xs}}$) arising from input from geological sources within or below the aquifer:

3 $${C}_\mathrm{i}^{\mathrm{meas}}={C}_\mathrm{i}^{\mathrm{atm}}\,+\,{C}_\mathrm{i}^{\mathrm{xs}}$$

${C}_\mathrm{i}^{\mathrm{atm}}$ is calculated for each sample using the CE model through PANGA^64^. It comprises both the equilibrium air–water component (that is, air-saturated water) and an excess air component that arises from dissolution of entrapped air bubbles during recharge^65^:

4 $${C}_\mathrm{i}^{\mathrm{atm}}={C}_{\mathrm{iw}}^{\;{\mathrm{eq}}}\times\left(\frac{1+{V}_\mathrm{a}/{V}_\mathrm{w}\times\,{H}_\mathrm{i}}{\,1+{V}_\mathrm{b}/{V}_\mathrm{w}\times\,{H}_\mathrm{i}\,}\right)$$

where *V*_a _*/* *V*_w_ is the initial air/water ratio in the recharge system and *V*_b _*/* *V*_w_ is the final bubble/water ratio as the water becomes isolated after recharge. *H*_i_ is the Henry solubility coefficient for noble gas i and is a function of temperature (*T*) and salinity (*S*)^66^. ${C}_{\mathrm{iw}}^{\;{\mathrm{eq}}}$ is the expected concentration of noble gas i based on equilibrium between the groundwater and atmosphere (that is, air-saturated water), defined via Henry’s law as^65^:

5 $${C}_{\mathrm{iw}}^{\;{\mathrm{eq}}}\left(T,S,{P}_\mathrm{a}\right)=\frac{{C}_{\mathrm{ia}}(T,{P}_\mathrm{a})}{{H}_\mathrm{i}(T,S)}$$

Where *C*_ia_ is the concertation of noble gas i in atmospheric air.

${C}_{\mathrm{i}}^{\mathrm{xs}}$ comprises accumulation of geological noble gas isotopes from both in situ production (${C}_{\mathrm{i}}^{\mathrm{in}\,\mathrm{situ}}$) within the aquifer and from deeper external sources (${C}_{\mathrm{i}}^{\mathrm{ext}}$), which may be mantle or crustal in origin:

6 $${C}_{\mathrm{i}}^{\mathrm{xs}}={C}_{\mathrm{i}}^{\mathrm{in}\,\mathrm{situ}}+\,{C}_{\mathrm{i}}^{\mathrm{ext}}$$

${C}_{\mathrm{i}}^{\mathrm{xs}}$ is determined for ^3^He, ^4^He and ^40^Ar by subtracting measured concentrations from atmosphere-derived concentrations (equation (3)). Then ${C}_{\mathrm{i}}^{\mathrm{ext}}$ is determined by subtracting an in situ ${C}_{\mathrm{i}}^{\mathrm{in}\,\mathrm{situ}}$ contribution (for ^4^He and ^3^He) from ${C}_{\mathrm{i}}^{\mathrm{xs}}$ through a Monte Carlo simulation framework to assess uncertainty in externally derived Ar and He isotope abundances. In each Monte Carlo simulation (*n* = 1,000), the amount of helium in groundwater that accumulates from in situ production $({C}_{\mathrm{i}}^{\mathrm{in}\,\mathrm{situ}})$ is calculated using the in situ accumulation rate (*P*_in situ_ in cm^3^ g^−1^ yr^−1^) determined from the purely crustal samples (main text) and the radiocarbon age (^14^C_age_ in years) of each sample:

7 $${C}_{\mathrm{i}}^{\mathrm{in}\,\mathrm{situ}}\,=\,{P}_{\mathrm{i}}^{\mathrm{in}\,\mathrm{situ}}\times\,{14\atop}\mathrm{C}_{\mathrm{age}}$$

We prescribe a Gaussian uncertainty for both ${P}_{\mathrm{i}}^{\mathrm{in}\,\mathrm{situ}}$ and ${14\atop}{\rm{C}}_{{\rm{age}}}$, separately, of 25% (1*σ*). For ${P}_{\mathrm{i}}^{\mathrm{in}\,\mathrm{situ}}$, this 25% uncertainty serves as a conservative estimate equal to twice the deviation (that is, ~12.5%) between the mean rate found in our analysis of the shallow crustal samples (1.49 × 10^−11^ cm^3^_STP_ g_w_^−1^ yr^−1^ and the previously published value for CRB helium production (1.7 × 10^−11^ cm^3^_STP_ g_w_^−1^ yr^−1^ (ref. ^34^)). Additionally, given that the ^14^C age only varies by a factor of three and considering that all of these samples are from within similar Columbia River Flood Basalt units, we would argue that it is reasonable to assume that the in situ helium production/accumulation rate is generally consistent. For ${14\atop}{\rm{C}}_{{\rm{age}}}$, the 25% uncertainty estimate accounts for potential biases in radiocarbon dating due to mixing or ^14^C-free DIC input, typically assumed to be on the order of several kyr. Notably, the impact of propagated errors on the subtraction of in situ helium in the deeper wells is a minor overall source of uncertainty in ^4^He_ext_ because the ^14^C ages of the deeper samples (ranging from ~15–24 kyr) are only a factor of approximately three larger than those of the shallow crustal samples (ranging from ~5 to 10 kyr), and ^4^He_in situ_ represents at most 25% of total ^4^He_xs_ among the deeper, mantle-influenced samples. Similarly, we prescribe Gaussian uncertainties in ${C}_\mathrm{i}^{\mathrm{xs}}$ that come from the quadrature sum of the measured values and CE model estimates of ${C}_{\mathrm{i}}^{\mathrm{atm}}$ (ref. ^24^). The Monte Carlo analysis results in mean values and uncertainties (which are propagated through from assumed values and model and measured uncertainties) for ${C}_\mathrm{i}^{\mathrm{ext}}$, which also is used to constrain the endmember mixing model, with uncertainties propagated throughout. The mixing model assumes that the external volatile source reflects a binary mixture between mantle-like and crustal-like endmembers. We constrain the model in ^3^He/^4^He vs ^4^He/^40^Ar space (Fig. 4 and Extended Data Fig. 5) using ${C}_\mathrm{i}^{\mathrm{ext}}$ values for ^40^Ar, ^4^He and ^3^He from the 13 deep wells via least squares:

$${\frac{{3\atop}\mathrm{He}}{{4\atop}\mathrm{He}}}_{\mathrm{ext}}=\,{\frac{{3\atop}\mathrm{He}}{{4\atop}\mathrm{He}}}_{\mathrm{crust}}\times f+\left(1-f\;\right)\times{\frac{{3\atop}\mathrm{He}}{{4\atop}\mathrm{He}}}_{\mathrm{mantle}}$$

$${\frac{{4\atop}\mathrm{He}}{{40\atop}\mathrm{Ar}}}_{\mathrm{ext}}=\,{\frac{{4\atop}\mathrm{He}}{{40\atop}\mathrm{Ar}}}_{\mathrm{crust}}\times f+\left(1-f\;\right)\times {\frac{{4\atop}\mathrm{He}}{{40\atop}\mathrm{Ar}}}_{\mathrm{mantle}}$$

Where *f* is the proportion of crustal derived fluids. The mantle and crustal endmember compositions were variable to best fit the data. Notably, the crustal ^4^He/^40^Ar must be highly fractionated compared to the conical value, however the model is insensitive to the actual value.

Whereas our model used a Monte Carlo framework (*n* = 1,000 simulations) to account for error propagation in the determination of excess and external helium and argon, only the mean results were used to fit the mixing curve (that is, Fig. 4). The purpose of this mixing model is to determine which, if any, of the known canonical mantle endmembers could explain the data, by evaluating the fitted curve over the known range of mantle ^4^He/^40^Ar production ratios (that is, the known range goes from ^4^He/^40^Ar of 1 to 3). Our analysis asks the question, what is the maximum plausible mantle ^3^He/^4^He associated with the minimum plausible ^4^He/^40^Ar (that is, ^4^He/^40^Ar = 1), and by fully propagated uncertainties on ^4^He_ext_/^40^Ar_ext_ and ^3^He_ext_/^4^He_ext_, we find the maximum mantle ^3^He/^4^He (associated with ^4^He/^40^Ar = 1) is 5.5 ± 0.4 R_A_ (1 *σ*). This allows us, from a more robust statistical perspective, to demonstrate the compatibility of an SCLM source and the incompatibility of a plume or MORB source of mantle helium.

### Potential subsurface tectonic driven devolatilization

Here we briefly explore the possibility that subsurface tectonic structures drive devolatilization. Two possible minor faults with minimal offset within the study region might exist^39^ (Extended Data Fig. 1). However, the basin is considered stable and lacks deformation^40,41,67^, and as a result we do not expect a substantial influence from movement along faults within the PBA. Although some studies have evidence of a minimal (~1%) slow velocity anomaly beneath the PBA^68^, which is very weak compared to other tomographic features within the region and probably has limited impact on volatile transport, however this anomaly has not been detected in other studies^69^. As a result, presently we cannot determine the cause of devolatilization and can only speculate a transport mechanism for these mantle volatiles. However, this does not impact our finding of non-volcanically active areas undergoing passive degassing of the SCLM.

### Evaluating prospective fractionation of the source

It is possible that the mantle ^4^He/^40^Ar ratio could be theoretically lower than the mantle production ratio (2 ± 1 (ref. ^8^)) as a result of degassing^8^, and lower ratios of ^4^He/^40^Ar (< 1) have previously been observed in SCLM-derived xenoliths^8,70^. As a sensitivity test (Extended Data Fig. 5), we consider the ^4^He/^40^Ar resulting from mixing curves using different mantle ^3^He/^4^He endmembers (MORB = ~ 8 and Yellowstone Plume = ~19 (refs. ^8,10–12^)). We find that a MORB-like mantle endmember ^3^He/^4^He would require a ^4^He/^40^Ar of ~0.7 and a plume-like Yellowstone ^3^He/^4^He endmember would require a ^4^He/^40^Ar of ~0.3, both of which are considerably below the canonical mantle production value, therefore requiring even more fractionation of the mantle. We also note that some fractionation may have occurred during the presently unidentified transport mechanisms. If diffusion-controlled fractionation occurred, this could increase the ^4^He/^40^Ar* as ^4^He is more mobile than ^40^Ar. We suggest that the conceptually simplest (given its intraplate location and lack of plume evidence) and most likely source of mantle-derived volatiles to the PBA is diffusive degassing of the SCLM.

## Online content

Any methods, additional references, Nature Portfolio reporting summaries, source data, extended data, supplementary information, acknowledgements, peer review information; details of author contributions and competing interests; and statements of data and code availability are available at 10.1038/s41561-025-01702-7.

## Source data

Source Data Source data files for Figs. 1–4, Extended Data Figs. 1–5 and Extended Data Tables 1–3.

## Figures and Tables

**Extended Data Table 1:**
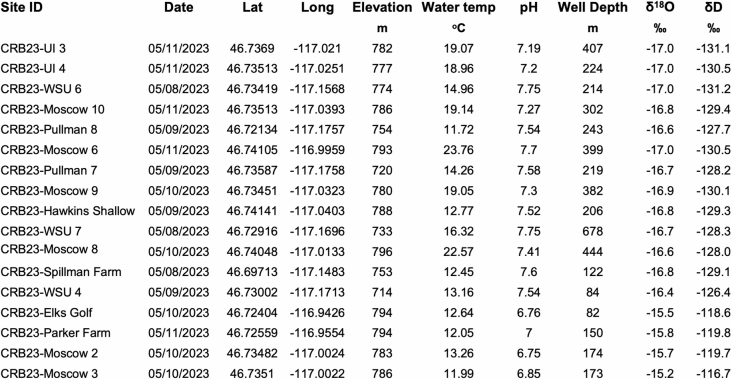
Sample location, site information, pH, temp and water isotopes

Source data

**Extended Data Table 2:**
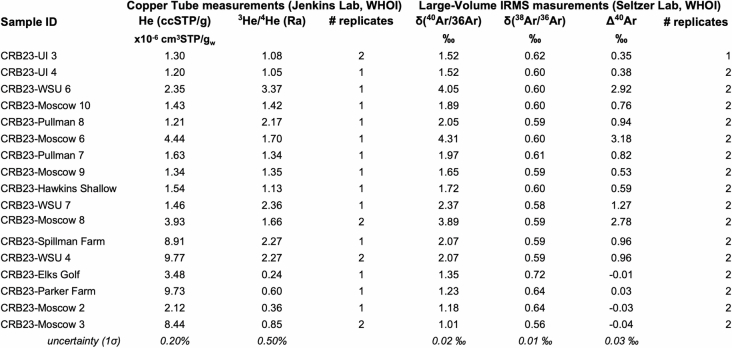
Measured helium and argon concentrations and ratios

All ratios are given relative to air.

Source data

**Extended Data Table 3:**
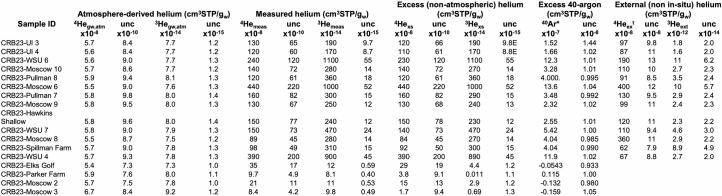
Measured, atmosphere derived, excess and deep external ^3^He and ^4^He concentrations and excess ^40^Ar concentrations

Uncertainties are 1σ and include measured and modelled where applicable.

Source data

## Data Availability

The geochemical data that support the findings of this study are available in the extended data tables (He and Ar isotopes) and via Zenodo at 10.5281/zenodo.12682511 (ref. ^24^). Source data are provided with this paper.
